# Evaluation of a Mass-Media Campaign to Increase the Awareness of the Need to Reduce Discretionary Salt Use in the South African Population

**DOI:** 10.3390/nu9111238

**Published:** 2017-11-12

**Authors:** Edelweiss Wentzel-Viljoen, Krisela Steyn, Carl Lombard, Anniza De Villiers, Karen Charlton, Sabine Frielinghaus, Christelle Crickmore, Vash Mungal-Singh

**Affiliations:** 1 Centre for Excellence in Nutrition (CEN), Faculty of Health Sciences, North-West University, Potchefstroom 2520, South Africa; 2 Chronic Disease Initiative for Africa, University of Cape Town, Private Bag X3 Observatory, Cape Town 7925, South Africa; krisela.steyn@uct.ac.za; 3 Biostatistics Unit, South African Medical Research Council, Tygerberg, Cape Town 7505, South Africa; carl.lombard@mrc.ac.za; 4 Non-Communicable Diseases Research Unit, South African Medical Research Council, Tygerberg, Cape Town 7505, South Africa; Anniza.deVilliers@mrc.ac.za; 5 School of Medicine, Faculty of Science, Medicine and Health, University of Wollongong, Wollongong, NSW 2522, Australia; karenc@uow.edu.au; 6 Illawarra Health and Medical Research Institute, Wollongong, NSW 2522, Australia; 7 MQ Market Intelligence, 5 Windward Turn, Atlantic Beach, Cape Town 7441, South Africa; sabine@mqmi.net; 8 Heart and Stroke Foundation South Africa, Unit 5B, 5th Floor, Graphic Centre, 5 Buiten Street, Cape Town 8001, South Africa; christelle@heartfoundation.co.za (C.C.); vash@heartfoundation.co.za (V.M.-S.)

**Keywords:** salt reduction, mass-media public health campaign, salt strategy

## Abstract

The South African strategic plan to reduce cardiovascular disease (CVD) includes reducing population salt intake to less than 5 g/day. A mass media campaign was undertaken to increase public awareness of the association between high salt intake, blood pressure and CVD, and focused on the reduction of discretionary salt intake. Community based surveys, before and after the campaign, were conducted in a cohort of black women aged 18–55 years. Questions on knowledge, attitudes and beliefs regarding salt use were asked. Current interest in engaging with salt reduction behaviors was assessed using the “stage of change” model. Five hundred fifty women participated in the baseline study and 477 in the follow-up survey. Most of the indicators of knowledge, attitudes and behavior change show a significant move towards considering and initiating reduced salt consumption. Post intervention, significantly more participants reported that they were taking steps to control salt intake (38% increased to 59.5%, *p* < 0.0001). In particular, adding salt while cooking and at the table occurred significantly less frequently. The findings suggest that mass media campaigns may be an effective tool to use as part of a strategy to reduce discretionary consumption of salt among the population along with other methods.

## 1. Introduction

Reducing mean population salt intake by 30% by 2025 is one of the World Health Organization’s (WHO) [1] global targets to reduce and control hypertension and non-communicable diseases (NCDs). In rural South Africans, stroke, the second most common cause of death and the leading cause of disability [2], is associated with high blood pressure and excess weight [3]. In their strategic plan for the prevention and control of non-communicable diseases (2013–2017), the South African National Department of Health includes the target to reduce the mean population intake of salt to less than 5 g per day [4]. Current salt intake by the South African population is well above this amount [5,6] with about 30% taking in more than 10 g of salt/day [6], as confirmed by a recent systematic review of the sub-Saharan Africa region [7].

To achieve these targets, two essential complementary strategies are needed. The first relates to changes to the food supply. It is estimated that approximately 60% of total salt intake is provided from processed foods, while the remaining 40% comes from discretionary use of salt in domestic food preparation and salt added to food during meals [8]. The South African government has adopted a legislative approach (implemented June 2016) that requires the food industry to comply with maximum targets for salt levels in a wide range of food categories, with a further reduction required by 2019 [9]. From the time that the Minister of Health initiated the national salt reduction strategy for South Africa, there have been a number of activities undertaken by the Department of Health and non-governmental organizations which have received wide coverage in the media. Examples include newspaper and magazine articles and TV airing that reported about the excessive salt intake of the population, promulgation of the salt reduction regulations and high level meetings held in the country on NCDs including the WHO/United Nations United Nations High-level meeting on NCD Prevention and control to shape the international agenda from 19 to 20 September 2011 in New York, USA.

The second strategy relates to efforts to change consumer behavior related to salt use. An advocacy group, Salt Watch (www.heartfoundation.co.za), that was formed in 2014 and funded, in part, by the National Department of Health through the Heart and Stroke Foundation South Africa (HSFSA), was mandated to run a mass-media campaign to increase public awareness related to the association between a high salt intake, blood pressure and cardiovascular disease, and to highlight the need to reduce discretionary salt intake. In addition, it also provided some information on which foods contain less salt. The campaign was based on sound behavior change principles [10,11] and the evaluation of the intended impact of the campaign was central to its initial planning. The progression through the different stages of change [12,13] was used as an outcome measure. The campaign supports the revised South African Food-Based Dietary Guidelines that state, as part of a healthy eating pattern, the population should “Use salt and foods high in salt sparingly” [5]. It consisted of television and radio advertisements as well as various supporting activities aimed at strengthening the advertisement message and providing additional information and education materials regarding salt reduction. The primary target audience were adult black women as the persons primarily responsible for food purchases and preparation in the household.

In this paper, we report on the baseline and follow-up survey after the intervention to assess the impact of a public awareness campaign by recording shifts after the intervention period, such as reach and participation, as well as changes in the knowledge, attitudes, beliefs and intended behaviors of the target audience.

## 2. Materials and Methods

### 2.1. Development and Implementation of the Public Awareness Campaign

A multi-sectorial group of salt reduction stakeholders formed an advocacy group called Salt Watch, which was launched during the Salt Summit meeting held in Johannesburg, South Africa on 13 March 2014. Salt Watch is led by the HSFSA and endorsed by the South African National Department of Health and was responsible for implementing the Public Awareness Campaign (for more information visit www.heartfoundation.co.za).

The campaign aimed to impact on several of the key processes described in the Theory of Reasoned Action (TRA) [10] to be instrumental in an individual deciding to change their behavior, namely: knowledge, attitudes, beliefs and intentions. In keeping with this theory, a successful salt awareness campaign will therefore require in the first instance an increase in knowledge about the dangers of high salt intake and then show improvements in the population’s attitudes to, and beliefs about the need to reduce high salt intake. Changes in these intermediary factors can be reasonably expected to increase the likelihood of an increase in the appropriate behaviors to reduce salt intake in the future.

The public awareness campaign consisted of two aspects. The main activity involved the development of one 30 s television advertisement and two 30 s radio advertisements for the most popular television channels and radio stations that are utilized by the target population. The two radio advertisements were translated into three commonly spoken languages in South Africa (English, isiXhosa and isiZulu) (six in total). The television advertisement was in English with isiXhosa subtitles and in isiZulu with English subtitles (two in total). These were accompanied by sign language in the case of the television advertisements. Thus a total of eight advertisements were developed (six radio and two TV). The advertisements featured a well-known South African medical doctor and media personality who emphasized the message that South Africans are consuming too much salt and that too much salt leads to hypertension, which can cause heart attacks and strokes. The doctor further urged South Africans to reduce their discretionary salt intake and ask their local clinic or doctor for more information. After extensive piloting of the content of the advertisement in the target population, the advertisements ran for a total of six months from 14 August 2014 to 24 May 2015 with an average of 44 television airings and 131 radio airings per month. The advertisements were not aired from beginning of December 2014 to end of February 2015 since this is the summer holiday period in South Africa and fewer listeners and viewers were anticipated.

The second aspect of the public awareness campaign included various supporting activities aimed at strengthening the advertisement message and providing additional information and education materials regarding salt reduction. The Salt Watch website (www.heartfoundation.co.za/), housed a mobile site function that utilized unstructured supplementary service data (USSD) technology that enabled the public to engage with Salt Watch in order to obtain more information and lower-salt recipes. Salt Watch brochures were developed and translated into five different languages for distribution, free of charge, and could be downloaded from the Salt Watch website. Distribution was implemented through various platforms, scientific congresses, symposiums, and brochures were provided upon request from the public or health care practitioners, as well as at free HSFSA public health screenings and HSFSA wellness days. Further awareness activities made use of the HSFSA infrastructure and existing media relationships to engage mass media to carry content provided through media releases (*n* = 8) at regular intervals throughout the campaign. These activities generated broadcast clips (*n* = 162), and yielded print and online articles (*n* = 195). The HSFSA further dedicated their social media platforms to salt reduction messaging and awareness during relevant periods of the campaign, including Facebook, twitter and their monthly newsletter titled Heart Zone. Through a partnership with a local pharmaceutical company, the HSFSA launched a second edition of their recipe book “Cooking from the Heart” which contained 36 reduced-salt recipes, in addition to general healthy eating information. The recipe book (*n* = 58,000 printed), which was only available in English, featured dedicated salt reduction messaging on six of its pages and was mainly distributed free of charge via health care professionals.

Health care professionals (dietitians, nutritionists, general practitioners, physicians, nurses and community health workers) were engaged during the campaign as they were identified as being highly influential stakeholders to provide salt reduction messages during health care interactions with individuals. Information regarding the Salt Watch campaign as well as educational tools and resources (e.g., patient brochures, a “Salt and Health” educational presentation with speaker notes, and a salt information manual) were shared with the identified healthcare professionals through various platforms. These included presentations and/or exhibitions at relevant congresses and meetings, a medical journal editorial [14], continued professional development articles (*n* = 2) for health care professionals and informative e-mails sent by professional associations/societies to their members. Table 1 provides detailed information on the number of individual health care professionals reached.

### 2.2. The Setting and Study Population

Community based surveys were conducted in a cohort of a convenience stratified sample of black women aged 18–55 years. A research agency, MQ (Market Intelligence) Market Intelligence of Cape Town was commissioned to undertake participant recruitment, obtain consent and administer the questionnaire within the participants’ homes using experienced fieldworkers. The surveys were conducted in urban areas of three South African provinces: Gauteng (14 suburbs); Eastern Cape (14 suburbs); and KwaZulu-Natal (19 suburbs). The selection of the provinces was informed by the campaign’s prime target market. In these provinces, consumers speak mainly isiXhosa or isiZulu. Within each suburb, there were predetermined quotas to ensure optimal representation of the women in the study areas. The number of participants per province was calculated based prorata on the number of people living in that province. Once this was known, the agency used a map to choose the specific towns and suburbs ensuring a wide geographical coverage of the province. The fieldworkers then looked for households that were willing to conduct an interview with them for the baseline as well as the after study. The fieldworkers also ensured that the interviewee has not been interviewed for at least three months and that she fits other recruitment criteria. In order to get a good spread of each of the three provinces the number of interviews was distributed over different areas. For example, not more than 5 households were interviewed in a specific area. The woman selected to be interviewed in each household were black women aged 18–55 years who self-identified as the main purchaser and decision maker of household food and groceries and were classified as meeting the South African Living Standards Measurement (LSM) category of 3–7 [15]. In addition, we also selected English, isiXhosa or isiZulu speaking women. LSM is a marketing segmentation tool developed by the South African Audience Research Foundation that divides the South African population into relatively homogeneous groups according to their ownership of major household appliances and their degree of urbanization using 29 variables as indicators [15]. South Africans 16 years and older are categorized into 10 LSM categories with LSM 10 indicating a high standard of living and wealth and LSM 1 reflecting extreme poverty. The sample was stratified according to Province, age, LSM group, gender, race, language and purchasing and decision making power. We did not control for education, family size, family stage or blood pressure status.

### 2.3. Data Collection

The data collection was coordinated by the HSFSA. The original English baseline and follow-up survey questionnaires were developed by the research team and translated into the vernacular language of each region. The interview was conducted by a trained interviewer in the home in a one-on-one situation. The data were captured in an English paper version of the questionnaire. The baseline survey included questions on the socio-demographic characteristics of the participants, their knowledge, attitudes, and beliefs as well as past and current behavior in relation to salt intake and its relationship to health. Both open ended and closed questions were included in the survey instrument. Questions were either prompted or unprompted.

A “stages of change” questionnaire based on the theoretical framework of Proshaska and DiClemente [12] was included. Questions related to participants’ current interest in engaging with salt reduction behaviors and included the following: “I am not interested in lowering salt in my diet” (pre-contemplation); “I have the intention of doing that within the next six months” (contemplation); “I have the intention of doing that within the next months”(preparation); “I have started lowering my salt intake during the last six months”(action); “I have already lowered my salt intake for longer than six months” (maintenance). The follow-up questionnaire used the same baseline questions and added questions related to the recall of the participant’s exposure to the television and radio advertisements, as well as specific questions related to aspects of the intervention program.

Baseline interviews were conducted between the 6 and 12 August 2014 and follow-up interviews between 11 May and the 4 June 2015 by teams of experienced multilingual trained interviewers who lived locally. Interviews were conducted in the language preferred by the respondent, which was predominantly either IsiZulu or IsiXhosa. Back checks were performed for a minimum of 25% of the respondents to verify the interviewing process, as well as the quota requirements for each suburb.

### 2.4. The Analyses of Data

Quantitative data sets of the baseline and follow-up surveys were computerized and merged. The marginal frequency distributions of the responses in each period were tabulated and compared, using logistic and multinomial regression models and taking into account the dependency of the within participant pre-post responses through a cluster variance specification at this level. Through this setup, all 550 women were included in all the inferential analysis. A logistic regression model for dropout was used to investigate the association of baseline demographic factors for this outcome. For one of the key outcomes, stage of change, the mediation of region, level of education, age group and LSM group was investigated. Region specific results for stage of change are reported as result of this analysis. A *p* value of ≤0.05 is considered a significant change in the categories studied.

A coding frame for the open ended questions was developed after data from 15% of the sample was extracted. The responses were categorized to identify main and sub-categories. These categories were all maintained in 2015. In 2015, we added a number of new codes of answers that were not mentioned in the 2014 study. Some of the open ended questions that related to correct knowledge about the impact of a high salt intake, and questions about the content of the advertisements were pre-coded. The open ended questions were analyzed by hand by trained coders according to the developed coding frame.

## 3. Results

### 3.1. Demographics

Five hundred fifty black females participated in the baseline study and 477 of those who participated in the baseline survey were included in the follow-up survey. Seventy-one of the baseline participants (43 from Gauteng province) could not be found or refused to participate and two women’s data was excluded as they reported different names to those provided at the baseline survey. The participants from Gauteng province (a highly urbanized area) indicated that they do not have time to complete the questionnaire again or the cell phone number provided was not in operation. This provided a response rate of 86.7%. The majority of the participants were IsiZulu speaking, lived in Gauteng, had 7–12 years of schooling, were in the LSM 5–7 category, and had children younger than six years of age. Nearly 40% of the women reported that they knew their blood pressure status (Table 2).

### 3.2. Exposure to the Intervention

During the follow-up survey, over 40% of the participants (*n* = 202) reported having heard, read, or seen any food and/or health related advertisement campaign in the last few months, compared to less than 20% at baseline (*p* < 0.0001), across all age and LSM groups. At baseline, 60.8% of the participants reported that they had seen/heard some sort of salt-related health information. However, the question did not identify specific media activities. In the follow-up survey more than three-quarter (77.8%) of the participants (*n* = 202) reported that they had seen the specific Salt Watch media campaign that included salt-related health information on TV or heard the messages on the radio. Table 3 shows the unprompted recall of the content of the campaign advertisements by those participants who had seen or heard the advertisements (*n* = 202). The most frequently recalled messages were that “too much salt is bad for your health” followed by “you should eat less salt”.

### 3.3. Changes in the Knowledge, Attitude or Beliefs and Intended Behaviors

The changes in knowledge, attitudes, beliefs and self-reported behaviors are shown in Table 4. Most of the indicators of knowledge, attitudes and behavior change show a significant move towards considering and initiating reduced salt consumption.

Significant increases were found for knowledge items that were related to high salt intake and its health outcomes. All these items mentioned were presented in both the radio and television advertisements. Many of the target population knew before the intervention that high salt intake is a risk for developing hypertension.

In terms of salt behavior, most participants thought that they consumed the right amount of salt both before and after the intervention. However, in general terms, after the intervention, the participants thought that it was important to reduce the amount of salt consumed.

Post intervention, significantly more participants reported that they were taking steps to control their salt intake. In particular, adding salt while cooking and at the table occurred significantly less frequently after the campaign than before.

Reported salt practices after the campaign indicated significant reductions in the amount of salt used in cooking and at the table, accompanied by a significantly higher use of herbs and spices. No significant changes were found in any of the other prompted salt reduction behaviors (see Table 5).

**Figure 1:**
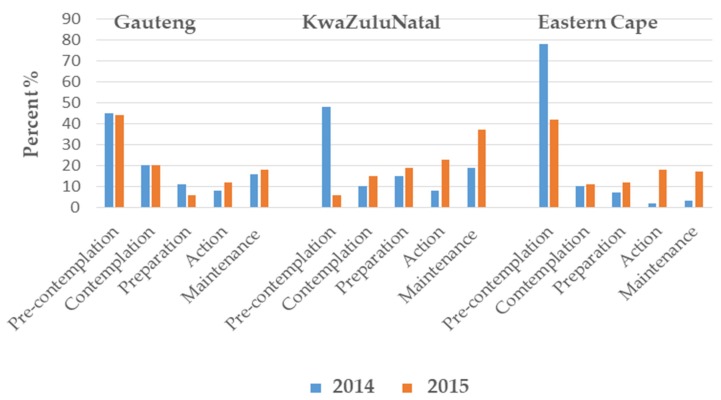
Percentage of participants in the various stages of change of salt consumption before and after the intervention in each region. The stages of change in salt consumption categories: pre-contemplation: no intention to reduce salt intake; contemplation: plans to reduce salt intake in the next 6 months; preparation: plans to reduce salt intake in the next month; action: reduction in salt intake has been initiated; maintenance: reduced salt intake has been maintained for 6 months.

Figure 1 illustrates the shift in the stages of change in salt consumption for the study. The multinomial logistic regression analyses identified a differential intervention effect (*p* < 0.0001) between the three regions with Gauteng showing no intervention effect (*p* = 0.2405) in contrast to the other two regions, KwaZulu-Natal and Eastern Cape, where a significant (*p* < 0.0001) shift towards initiation of salt reduction was observed (*p* < 0.0001).

## 4. Discussion

The evaluation demonstrated the effectiveness of a public health awareness campaign to increase knowledge and awareness of the health consequences of a high salt diet in South African black women of low-middle socioeconomic status. Our data also suggested shifts in salt behaviors in some of the target population. This positive change among black women to considering a reduction in their discretionary salt intake has not previously been achieved in other low and middle income countries through the use of a mass media public health awareness campaign. This is particularly important in this setting as South Africans consume about 40% of their salt from discretionary sources [8]. One of the staple foods in South Africa is homemade maize meal and consumers tend to add soup powder, stock cubes and/or a monosodium glutamate-based flavoring (all are high in salt) for a change in taste. Furthermore, South Africans, being from a low income country, probably eat less processed foods than consumers in high-income countries. This is, however, likely to change. From 1999 to 2012, there has been an increase in the consumption of processed foods and beverages (soft drinks, sauces, dressings and condiments, and sweet and savory snacks), which is typical of an upwardly mobile consumer population [16]. This contrasts with the situation in the United Kingdom where discretionary salt intake provides a minor contribution to overall salt intake (15%) [17].

We showed a significant shift to lower reported usage of salt at the table as reflected in increased reporting of “rarely” and “sometimes” in 2015 compared to 2014. This could be explained by our finding of significant improvement in the salt knowledge of the participants from 2014 to 2015. The improved knowledge reflected the content of the television and radio advertisements. Nearly a third of the participants reportedly had been diagnosed with hypertension, which may explain why there was a relatively high knowledge at baseline that “a high salt intake is bad for your health” (75.5%) and “a high salt intake is related to developing hypertension” (75%).

In this study, 59% of participants at follow-up, on direct questioning, reported not adding salt while cooking. This is much higher than reported in an Australian cross-sectional study where 35.1% of the participants reported this behavior [18] which was similar to reported behavior at baseline in our cohort. In addition, we found a significant shift to lower usage of salt at the table as reflected in increased reporting of “rarely” and “sometimes” in 2015 compared to 2014. This again suggests the positive impact of our awareness campaign. In the United Kingdom, one of the contributing factors to the success of efforts to reduce population-level salt intake [19] through their national salt reduction campaign, is a reduction in the practice of adding salt at the table [20]. The difficulty in showing an association between reported salt-related behavior change and biological outcomes is illustrated by a recent Australian study, which found no evidence of an association between any measure of knowledge, attitudes or behaviors and a single 24-h urinary salt excretion (before or after adjustment for age, gender, body mass index and highest level of education) [21]. In our study, we did not investigate the association between actual salt intake using biomarkers and the categorization of individual stages of change. However, the High-Risk and Population Strategy for Occupational Health Promotion (HIPOP-OHP) Study [22] demonstrated a significant association between stage of change for reported dietary salt intake behavior and spot urinary sodium excretion for both males and non-obese females. In that study, those with in the pre-contemplation stage had a higher salt intake than those classified as being at a later stage of change. The unprompted reporting of the participants of the actual content of the advertisements also strongly suggests that the discretionary salt reduction campaign resulted in a reduced discretionary salt use by the target population. The finding that there was a significant positive shift in participants reporting increasing use of herbs and spices, rather than salt in food preparation, is also very encouraging. These actions to reduce salt intake were not included in the content of the television and radio adverts but only in the additional materials that was made available by Salt Watch. This reflects the increased impact of the use of multimedia in conveying health messages to a study population and suggests that such an approach should be used whenever feasible in population-based health campaigns.

It is encouraging that there were positive shifts in the stages of change categories towards reducing salt intake, at least in two of the three provinces sampled, as this holds promise that the campaign achieved its goals (Figure 1). Possible reasons why Gauteng participants did not demonstrate a significant shift in their stage of change may be related to a greater degree of urbanization compared to the other two provinces, or that these residents may have been more exposed to general salt reduction messages before the beginning of the mass media campaign. A combined analysis using the multinomial logistic regression identified this shift towards initiation of salt reduction to be significant (<0.001) (data not shown).

Furthermore, these increased desirable actions were also confirmed by the significant reported shifts towards less discretionary salt use. The data also illustrates that changes in the initial steps described in the Theory of Reasoned Action of behavior change occurred in our study population; knowledge improved and beliefs and attitudes shifted towards the desired steps that may lead to less salt use in future [10]. In addition, a previous multi-country survey on the stages of change regarding salt reduction in high LSM groups (internet access and email address were inclusion criteria) reported that about 55% of the studied group (in South Africa) of men and women were in the first three stages combined [23]. It thus seems as if the lower LSM groups moved to a similar pattern of the stages of change in 2015 (first three stages combined) as compared to the higher LSM groups in the multi-country study published in 2013. Participants were from Brazil, India, China, Germany/Austria, Hungary and United States of America.

The World Health Organization has clearly identified a 30% salt reduction as one of the essential targets to control NCDs. It has also been identified of one of the “Best Buys” that countries can undertake to achieve reduced NCD burden [24]. The South African government has initiated its salt policy activities by formulating salt reduction regulations [9] and by supporting the Salt Watch led salt awareness campaign. This evaluation of a media campaign and additional supported activities of Salt Watch indicates that the strategy holds promise to reduce discretionary salt intake in South Africa, particularly if the health promotion components become incorporated as part of an ongoing health promotion campaign for the country.

### 4.1. Limitations

There are limitations to our study. The study has limited generalizability as the study population included only black women from urban settings from low to middle living standards measures. The follow-up survey was conducted soon after the end of the airing of the advertisements and it cannot be assumed that the impact of the salt reduction messages would be maintained over the longer term. It would have been useful to re-evaluate the participants some months after the population-based intervention was completed to assess how frequently such campaigns would need to be repeated to ensure long term maintenance of the messages. In addition, the same individuals were included in the baseline and follow-up surveys. They could have been prompted to investigate the facts surrounding salt consumption as a direct result of taking part in the study, hence influencing your findings during the follow-up survey.

The respondents in the study were selected as a convenience sample in 47 suburbs of the selected three provinces. Thus although the study has some regional representativeness it is not based on any formal sampling frame. The statistical inference conducted therefore assumes a random sample from the target population. The significance of the results reported is therefore only illustrative under this required assumption.

The same people were interviewed in 2014 and 2015 thus resulting in potential reporter bias because of social desirability as reported in the literature [25]. The lack of a control group is a major limitation to the study design but could not be included because of the population level intervention strategy which necessitated a pre-post quasi experimental study design. It was not possible to use, for example, a neighboring country as a control group since no information is available on the usual eating habits, salt intake or existing salt reduction strategies of neighboring southern African countries.

Theory of Reasoned Action (TRA) [10] suggests that a person’s behavior is determined by his/her intention to perform the behavior. This intention is predicted by their knowledge, beliefs and attitudes regarding a specific behavior, and by their beliefs about whether individuals who are important to them approve or do not approve of the behavior (subjective norm). In this paper, we did not interrogate the last aspect of the TRA.

Blood pressure status was not ascertained during the study and the possible association with baseline knowledge could not be adjusted for the analysis.

### 4.2. Possible Additional Research

Additional research is needed on the stages of salt change. Since the Australian study [21] did not investigate the association between stages of salt change and salt intake as measured by 24-h urine excretion (gold standard method), it would be worthwhile to do so. Collection of 24-h urinary samples in order to measure salt intake is expensive and logistically complex to conduct on a population level. If an association between salt intake (using 24-h urinary samples) and stages of behavior change could be demonstrated, the use of the stages of behavior change could be a less invasive method of evaluating salt intake of a population.

## 5. Conclusions

The success of a public health campaign that aimed to improve population-level knowledge and attitudes related to discretionary salt use has been demonstrated in black South African women from low income settings. However, the findings should be extrapolated to other groups with care. Significantly more participants reported that they were taking steps to control salt intake following the intervention. In particular, reported behaviors of adding salt to food while cooking, and at the table occurred significantly less frequently. The findings suggest that mass media campaigns may be an effective tool that can make an important contribution to strategies to reduce the consumption of discretionary salt intake among the South African population. Trieu and colleagues [25] in a recent review concluded that education or awareness campaigns alone are unlikely to be adequate to achieve the WHO target of a 30% reduction in average salt intake. We agree that multi-pronged strategies using multiple channels of communication need to accompany the salt reduction regulations of certain foods stuffs.

## Figures and Tables

**Table 1:** Information regarding the Salt Watch campaign shared with health care professionals.

Type of Information	General Medical Practitioners	Nurses	Dietitians/Nutritionists	Community Health Workers	Totals
Talks	30	30	1250	95	1405
Stand Interactions (Questions filled in)			92		92
Brochures	450		952	50,000	51,402
Emails			1717		1717
Editorials	2180	160,315	400		162,895
CPD			400		400
**Total**	**2660**	**160,345**	**4811**	**50,095**	**217,911**

CPD: Continuous professional development.

**Table 2:** Characteristics of participants in the baseline survey (*n* = 550).

Characteristics	Characteristics	Participants (*n*)	Participants (%)
Age group	18–35 years	276	50.2
	>35–55 years	274	49.8
Province	Gauteng	212	38.6
	KwaZuluNatal	208	37.8
	Eastern Cape	130	23.6
LSM group	LSM 3–4	155	28.2
	LSM 5–7	395	71.8
Education	Less than 7 years schooling	20	3.6
	Seven to 12 years schooling	375	68.2
	Any tertiary education	151	27.5
Family stage	No children	78	14.2
	With children under 6 years	231	42.0
	With children 7–12 years	139	25.3
	With children 13–18 years	71	12.9
	With children older than 18 years	28	5.1
Language	English	2	0.4
	IsiXhosa	138	25.1
	IsiZulu	392	71.3
Blood pressure (BP) status	Person knows their BP value	210	38.2
	Has been informed by a doctor or nurse? that they had high BP	112	20.4
	Currently taking BP medication	62	11.3

**Table 3:** Unprompted recalls by participants who reported having seen or heard the advertisements of the content of the advertisements during the follow-up survey (*n* = 202).

Open Ended Question Answers	Participants (%) *n* = 202
The food you buy already contains salt	9
Salt was poured on the table (TV image shown) and information given about how much salt we should use each day	15
You should use less salt	17
South Africans eat too much salt every day	24
High blood pressure can cause heart attacks and strokes	25
Too much salt can lead to high blood pressure	28
Too much salt is bad for your health	64

Besides the television and radio advertisements, participants (*n* = 143) also recalled health information on posters at clinics/hospitals (20%), in magazines (7%), by word of mouth (5%), general practitioner/dietitian (2%), and newspaper (1%).

**Table 4:** Knowledge, attitude, beliefs and self-reported behavior of participants regarding salt use before and after the campaign.

Questions	Baseline 2014 (*n* = 550) Participants (%)	Follow-Up 2015 (*n* = 477) Participants (%)	*p*
**Knowledge**			
High salt intake is bad for your health (when directly prompted)	75.5	89.4	≤0.0001
High salt intake is related to suffering strokes (unprompted)	8	50	Not available
Salt intake is related to heart disease (unprompted)	28	59	Not available
High salt intake is related to developing hypertension (unprompted)	75	77	Not available
**Attitudes and beliefs**			
Consume just the right amount of salt	74.4	71.2	0.609
It is very important to lower the salt in your diet	66.6	74.5	<0.001
**Behavior**			
Confirmed that they are controlling their salt intake	38.0	59.5	<0.0001
**Add salt at the table**			
Rarely	22.9	27.1	0.0015 ^1^
Sometimes	22.6	30.3	
Often	11.5	9.6	
Always	20.6	15.0	
**Add salt when cooking**			
Rarely	6.2	11.3	<0.0001 ^2^
Sometimes	10.4	25.3	
Often	17.5	20.0	
Always	63.3	40.3	

Not available: Multinomial regression analyses were not done on this unprompted data. ^1^ A significant change overall, *p* = 0.0015. A significant shift to lower usage of salt at the table as reflected in increased reporting of “rarely” and “sometimes” in 2015 compared to 2014. ^2^ A significant change overall, *p* < 0.0001. A significant shift in using salt in cooking to “rarely”, “sometimes” and “often” relative to “always”.

**Table 5:** Percentage of respondents before and after the intervention who reported changing their salt consumption behavior on direct questioning.

Salt Consumption Behavior	Baseline 2014 (%) *n* = 209	Follow-Up 2015 (%) *n* = 285	*p*
Avoid/minimize consumption of processed foods	64.2	64.2	0.992
Look at the salt or sodium labels on food	54.2	56.1	0.571
Avoid adding salt at table	14.2	20.1	0.049
Buy low **salt** alternatives	61.0	59.6	0.708
Buy low **sodium** alternatives	52.9	54.7	0.594
Do not add salt when cooking	45.2	59.1	<0.0001
Use herbs or spices other than salt when cooking	70.0	77.8	0.023
Avoid eating out	45.2	45.8	0.594
